# Factors contributing to therapeutic effects evaluated in acupuncture clinical trials

**DOI:** 10.1186/1745-6215-13-42

**Published:** 2012-04-21

**Authors:** Guang-Xia Shi, Xiao-Min Yang, Cun-Zhi Liu, Lin-Peng Wang

**Affiliations:** 1 Acupuncture and Moxibustion Department, Beijing Hospital of Traditional Chinese Medicine affiliated to Capital Medical University, 23 Meishuguanhou Street, Dongcheng District, Beijing, 100010, China

**Keywords:** Acupuncture, Clinical trial, Factors, Therapeutic effects

## Abstract

Acupuncture treatment has been widely used for many conditions, while results of the increasing numbers of randomized trials and systematic reviews remain controversial. Acupuncture is a complex intervention of both specific and non-specific factors associated with therapeutic benefit. Apart from needle insertion, issues such as needling sensation, psychological factors, acupoint specificity, acupuncture manipulation, and needle duration also have relevant influences on the therapeutic effects of acupuncture. Taking these factors into consideration would have considerable implications for the design and interpretation of clinical trials.

## Introduction

Acupuncture is a core component in traditional Chinese medicine (TCM) and can be traced back more than 3000 years in China [1]. The World Health Organization endorses acupuncture for at least two dozen conditions [2]. The US National Institutes of Health issued a consensus statement proposing acupuncture as a therapeutic intervention for complementary medicine [3].

The randomized controlled trial (RCT) is considered the gold standard to provide evidence for a treatment’s efficacy [4,5]. In recent years, there have been increasing numbers of RCTs showing that acupuncture was no more effective than sham acupuncture in migraine [6,7], low back pain [8,9], and knee osteoarthritis [10,11], although both interventions were more effective than no treatment. Sham acupuncture includes needle insertion at non-acupuncture points, shallow needle insertion that does not penetrate below the skin (minimal or superficial needling), and blunt needles that touch, but do not penetrate the skin (placebo needling) [12].

The trial design comparing true and sham acupuncture presupposes that needling alone is the characteristic treatment element. However, acupuncture is a complex intervention of both specific and non-specific factors associated with therapeutic benefit. The lack of difference between real and sham acupuncture in RCTs may result from the omission of important components of acupuncture. As confounders in randomized designs, non-specific components of acupuncture maybe lead to false-negative results [13].

### Needling sensation

Needling sensation is considered by many acupuncturists to be an important component of acupuncture since the early classical texts [14]. A key term that relates to needling sensation is Deqi [15], a composite of unique sensations interpreted as the flow of Qi or ‘vital energy’. Based on the theory of TCM, acupuncture is successful only with the experience of Deqi, which suggests correct localization of the acupuncture point, and the arrival of Qi. This state is essential to acupuncture’s therapeutic effect although few data are available [16,17]. The process of drawing Qi with needles is experienced by both patient and acupuncturist. Patients experience Deqi as multiple unique sensations at the needle site itself and around the site of needle manipulation, including soreness, aching, numbness, tingling, and even warmth [18]. Simultaneously, acupuncturists feel a change in the mechanical behavior of the tissues surrounding the needle (needle grasp). This change is described as tense, tight and full like ‘a fish biting on a fishing line’ as described in the literature [19]. Needle grasp is a biomechanical phenomenon of Deqi, which was characterized by an increase in the force necessary to pull the needle out of the tissue (pullout force) [20]. Langevin’s trial supports connective tissue winding as the mechanism responsible for the increase in pullout force induced by needle rotation [21].

Experimental studies have found that Deqi sensation produces greater local blood flow at the needle site compared to simple needle insertion [22] and, the intensity of different acupuncture sensations are associated with subsequent analgesia. Kong et al. [23] found a similar relationship between acupuncture analgesia and numbness and soreness, but not for other sensations commonly associated with Deqi. A noticeable difference of therapeutic efficacy existed in some trials between acupuncture and sham acupuncture, in most cases because the latter did not involve manual stimulation and an attempt to induce Qi [24].

So far, the identification and investigation of these sensations have been widely ignored, mainly due to the lack of methodological consideration [25]. The sensory component of Deqi is difficult to study because of its subjective nature and it is influenced by many factors, such as the constitution of a patient, severity of the illness, location of the acupoints, and the needling techniques. There appears to be a limit to the number of sensations that can be discriminated by each individual patient [26]. A number of researchers have sought to establish a credible rating scale for Deqi, such as the McGill Pain Questionnaire [18,27], Subjective Acupuncture Sensation Scale [25], the Massachusetts General Hospital Acupuncture Sensation Scale [23], the Southampton Needle Sensation Questionnaire [28], and the ‘Deqi composite’ [29]. Although Deqi may have been traditionally intended to designate both the acupuncturist’s and patient’s perceptions [18], most of these scales have chosen to measure only one. Thus, existing measures of needling sensation are flawed and an improved questionnaire is essential to advance this area of research. Deqi should be taken into account in clinical trials and, further research is required to understand the underlying mechanisms [30].

### Psychological factors

Expectancy, a crucial component of psychological factors, plays an important role in acupuncture practice [31,32]. Clinical experience suggests that patients receiving acupuncture have to expect to be treated. The insertion of needles for diagnostic reasons seldom seems to gain good effect. One possible basis for acupuncture effects might thus be changes in the direction of patients’ attention. Positive expectation can significantly amplify acupuncture analgesia effects as evidenced by decreased subjective pain sensory rating as well as objective fMRI signal changes in response to calibrated noxious stimuli [33]. Diminished positive expectation appeared to inhibit acupuncture analgesia. Linde et al. [34] suggested that expectation of relief was the only factor that correctly predicted outcome, by reanalyzing results from several RCTs of acupuncture treatment for chronic pain. Patient’s expectation and belief regarding a potentially beneficial treatment modulate activity in component areas of the reward system [32]. Negative cognition resulted in a change of the relationship between the pre-experimental expectancy of acupuncture and self-reported pain. The negative group produced an increased low-frequency component of heart rate variability (HRV) after acupuncture, whereas the positive group did not [25].

A systematic review revealed that there have been relatively few research studies assessing the relationship between expectancy and treatment responses following acupuncture, and suggested future studies should both manipulate and assess expectancy of patients [35]. It could be used as a stratification factor in clinical trials of acupuncture. Recently, a mixed-methods approach in a single report conducted by White et al. indicated that patients’ beliefs about treatment veracity and confidence in outcomes were reciprocally linked, and this appears to shape how patients self-report outcome, complicating and confounding study interpretation [36].

In addition, psychological factors such as anxiety level should be considered as having an important influence on physiological response to acupuncture. It seems possible that the level of anxiety can modify HRV during acupuncture treatment and up to 40 min after the treatment [37]. A better understanding of the psychosocial factors involved in acupuncture should be able to improve outcomes for patients.

### Acupoint specificity

According to the theory of TCM, acupoint specificity is the important basis of clarifying the functionality of acupoints and guiding acupuncture clinical practice. Classic theory recognizes about 361 points. All were said to be located on 14 main channels (or meridians) connecting the body in a web-like interconnecting matrix [38]. Traditionally, each acupuncture point has defined therapeutic actions. Questions about point specificity in acupuncture thus remain open. The selection and compatibility of acupoints are considered to have direct impact on the therapeutic effect. Data from neuroimaging studies in humans suggest that modulation of the limbic system may differentiate between specific and non-specific components of acupuncture [39].

### Acupuncture manipulation

There are some methods of reinforcing-reducing manipulations of acupuncture in TCM, by slow and quick manipulation of needle, and manipulating the needle in cooperation with the patient’s respiration. They are usually applied in combination [40]. All manipulations are based on the principle of puncturing along and against the flowing direction of the meridian Qi. Mastering the reinforcing-reducing manipulations of acupuncture will contribute to the improvement of therapeutic effects in clinical practice [41]. Differences of reinforcing and reducing acupuncture manipulations have different influences on the activities of natural killer cell, lymphokine-activated killer, and T lymphocyte subsets in peripheral blood of malignant tumor patients [42]. In addition, lifting-thrusting and twirling-rotating are different technical processes; both of them could promote the flow of Qi, acting as reinforcement and reduction effects to a different extent. There are also variations in the direction, angle, and depth of needle insertion. With these factors combined, it is possible to affect the outcomes of acupuncture treatment [43,44].

### Needle duration

Needle duration refers to holding the needle in the acupoint after the use of needling manipulation, with the aim to wait for Qi arrival, regulate Qi circulation, eliminate pathogenic factors, and reinforce the antipathogenic Qi as well as the effect of manipulations. Different times of needle retention should be applied for different diseases [40].

Evidence from both human and animal studies has indicated that a striking feature of acupuncture analgesia is its longevity (a delayed onset), gradual peaking, and gradual returning [45]. For a typical 30-min acupuncture session, the pain threshold has a slowly increasing tendency even outlasting the treatment [46]. It is shown that the acesodyne effect could only be received when needle retention lasted for more than 20 min [47]. The effect of simple retention without manipulations for 15 min and 30 min were better than quick needling for 45 min in 289 cases of acute and chronic soft tissue injury [48]. The effect of 1 h of needle retention of body acupuncture might be better than 30 min in the cases of intractable hiccup [49]. Bao et al. observed the effect of 20 min, 40 min, and 60 min needle retention in the treatment of ischemic stroke, indicating there was a certain relation between retention duration and therapeutic effect. The longer the retention duration, the more significant the improvement detected on the myodynamia and hemorheology index [50]. However, quick needling could significantly relieve pain in systremma when compared to 30 min of needle retention [51]. Robust evidence of RCTs is needed to lend support for the effect of needle duration on acupuncture treatment.

### Other aspects

All complex therapeutic interventions achieve their outcomes through a combination of specific and non-specific mechanisms [52]. It is important to emphasize that acupuncture is not a simple needling intervention. Paterson and Dieppe argue for a more holistic experience in acupuncture than from needles alone [5]. Consistent with them, Kaptchuk contends that the therapeutic context for acupuncture depends not only on the specific therapeutic ritual applied but also on experiences, attitudes, and preferences of patients and providers, the patient-provider interaction, the setting, and the cultural background [15]. Previous trials that assessed the reliability of retractable type sham needling among acupuncture-naıve subjects or subjects with limited acupuncture experience revealed that patient’s knowledge and experience of acupuncture also account for the different results [53,54]. Close interaction between patient and therapist is typical for acupuncture and will often involve suggestive components. There is evidence that the same acupuncture intervention can have quite different effects when provided in different contexts [55]. Factors related to therapeutic relationship, in particular patients’ perceptions of that relationship, are likely to be important in facilitating good clinical outcomes [56]. Acupuncturists’ skills, competence, and understanding of the TCM theory, lifestyle advice, and even individualized treatment protocols also play important roles in the therapeutic outcome [57-59].

In addition, the diagnostic process plays an important role in therapeutic outcome. The Western diagnosis is the theoretical basis for prescribing the drug and standardized administration of the drug follows, and therefore occurs before the intervention is started. TCM diagnosis, however, through questioning and examination, will be made during the first treatment session and will be reviewed and amended at each subsequent session. In acupuncture practice, during subsequent treatment sessions repeated pulse-taking and feedback about the effects of needle insertion are often varied to take into account any new concerns. Subsequently, this may result in a different therapeutic method [5].

## Conclusions

In recent years, although there has been increasing evidence from large randomized trials and systematic reviews on the efficacy of acupuncture, the conclusions are controversial. The lack of difference between real and sham acupuncture in RCTs may result from the omission of important components of acupuncture. Assessing individual components will underestimate the true efficacy of acupuncture [36]. Apart from needle insertion, issues such as needling sensation, psychological factors, acupoint specificity, acupuncture manipulation, and needle duration also have relevant influences on the therapeutic effects of acupuncture. There may also be an interaction between different components within acupuncture treatment producing a clinical effect that is greater than the sum of its individual elements. Taking all these factors into consideration would have considerable implications for the design and interpretation of acupuncture clinical trials. Approaches such as factorial experiments, randomized pragmatic designs, and randomized cluster designs may be more appropriate and rigorous to sort out the complexity of acupuncture.

## Abbreviations

TCM, Traditional Chinese medicine; RCT, Randomized controlled trial; HRV, Heart rate variability.

## Competing interests

The authors declare that they have no competing interests.

## Authors’ contributions

SGX wrote and revised the manuscript; LCZ and WLP developed the original concepts for the review; YXM wrote the first draft of the paper. All authors contributed to the paper during development and read and approved the final version of the manuscript.
